# 369. Antibiotic Stewardship at a New York City Public Hospital NICU using Neonatal Early-Onset Sepsis Calculator in the Presence of Maternal Chorioamnionitis

**DOI:** 10.1093/ofid/ofae631.110

**Published:** 2025-01-29

**Authors:** Gabriela A Rodas Cedeno, Adila Chamavaliyathil, Jean-Marco Verdi, Jairus Flores, Lawrence Noble, Thaina Rousseau-Pierre, Jennifer Pintiliano, Uday Patil

**Affiliations:** Baylor College of Medicine at Texas Children's Hospital, Houston, TX; Icahn School of Medicine at Mount Sinai, Queens, New York; Icahn School of Medicine at Mount Sinai, Queens, New York; Icahn School of Medicine at Mount Sinai, Queens, New York; Icahn School of Medicine at Mount Sinai, Queens, New York; New York City Health and Hospitals, Queens, New York; Icahn School of Medicine at Mount Sinai, Queens, New York; Icahn School of Medicine at Mount Sinai, Queens, New York

## Abstract

**Background:**

Maternal chorioamnionitis is a major risk factor for neonatal early-onset sepsis (EOS) driving the use of empiric antibiotics in the neonatal intensive care unit (NICU). Utilization of the neonatal EOS calculator can guide the management and decrease antibiotic usage. Our objective is to safely reduce the exposure to empiric antibiotics (Ampicillin and Gentamicin) by 30% in a period of 6 months using the neonatal EOS calculator in full-term infants admitted to NICU in the setting of maternal chorioamnionitis at our public hospital.

Driver Diagram

**Figure:**
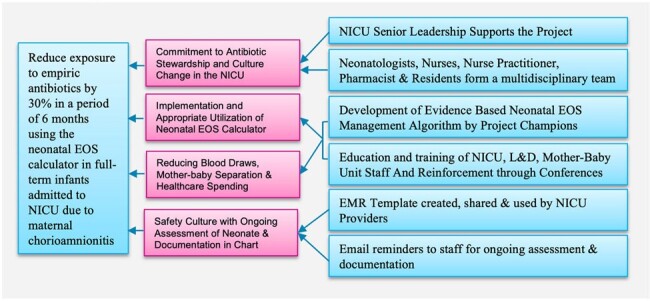

**Methods:**

Using the Model for Improvement framework, we developed a management algorithm with the neonatal EOS calculator along with documentation and workflow changes. A multidisciplinary team implemented PDSA cycles to promote adherence to the algorithm using education, training, and reinforcement. Baseline antibiotics usage rate (AUR) was calculated using chart reviews in the pre-quality improvement (QI) period (January 2023-July 2023).

Antibiotic Usage Rate (AUR) for PDSA 1 and PDSA 2

**Figure:**
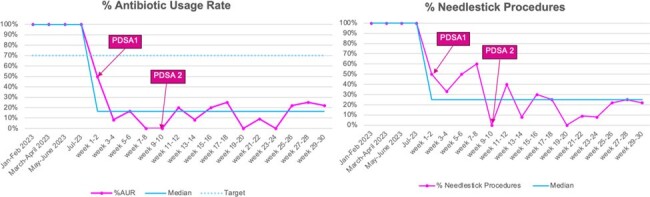

**Results:**

During the QI period (August 2023-February 2024) a total of 127 at-risk full-term neonates were managed using the neonatal EOS calculator. Of these, only 18 neonates received empiric antibiotics. When compared to the baseline AUR, this was a significant decrease by 85.8%. Additionally, the rates of laboratory investigations (blood culture and complete blood count) also showed significant decrease by 77% in this cohort with no positive blood cultures.

EOS Workflow Algorithm

**Figure:**
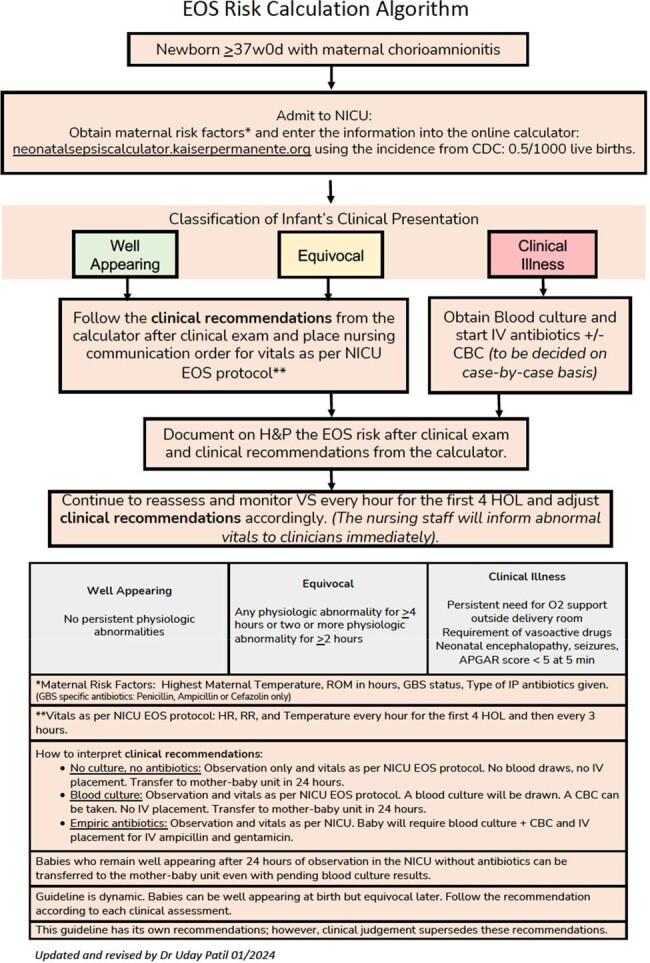

**Conclusion:**

Our QI study demonstrates that the implementation of neonatal EOS calculator-based management in full-term infants born after maternal chorioamnionitis is safe and can be highly effective in establishing antibiotic stewardship in the NICU at a public hospital.

**Disclosures:**

**All Authors**: No reported disclosures

